# *Panax ginseng* Fraction F3 Extracted by Supercritical Carbon Dioxide Protects against Oxidative Stress in ARPE-19 Cells

**DOI:** 10.3390/ijms17101717

**Published:** 2016-10-13

**Authors:** Chao-Chin Yang, Chiu-Yuan Chen, Chun-Chi Wu, Malcolm Koo, Zer-Ran Yu, Be-Jen Wang

**Affiliations:** 1 Department of Food Science, National Chiayi University, 300 Syuefu Road, Chiayi City 60004, Taiwan; yccn1324@gmail.com; 2 Department of Natural Biotechnology, Nanhua University, Dalin, Chiayi 62249, Taiwan; chiuyuan@mail.nhu.edu.tw; 3 Research and Extension Center of Natural Healing Sciences, Nanhua University, Dalin, Chiayi 62249, Taiwan; 4 Institute of Medicine, Chung Shan Medical University, Taichung City 40201, Taiwan; daniel@csmu.edu.tw; 5 Department of Medical Research, Chung-Shan Medical University Hospital, Taichung City 40201, Taiwan; 6 Department of Medical Research, Dalin Tzu Chi Hospital, Buddhist Tzu Chi Medical Foundation, Dalin, Chiayi 62247, Taiwan; 7 Dalla Lana School of Public Health, University of Toronto, Toronto, ON M5T 3M7, Canada; 8 Superwell Biotechnology Corporation, Taichung City 40876, Taiwan; zerranyu@gmail.com

**Keywords:** *Panax ginseng*, supercritical carbon dioxide fractionation, retinal pigment epithelium, hydrogen peroxide-induced oxidative damage

## Abstract

In our previous work, the ethanolic extract of *Panax ginseng* C. A. Meyer was successively partitioned using supercritical carbon dioxide at pressures in series to yield residue (R), F1, F2, and F3 fractions. Among them, F3 contained the highest deglycosylated ginsenosides and exerted the strongest antioxidant and anti-inflammatory activities. The aim of this study was to investigate the protective effects of *P. ginseng* fractions against cellular oxidative stress induced by hydrogen peroxide (H_2_O_2_). Viability of adult retinal pigment epithelium-19 (ARPE-19) cells was examined after treatments of different concentrations of fractions followed by exposure to H_2_O_2_. Oxidative levels (malondialdehyde (MDA), 8-hydroxydeoxyguanosine (8-OHdG), and reactive oxygen species (ROS)) and levels of activity of antioxidant enzymes were assessed. Results showed that F3 could dose-dependently protected ARPE-19 cells against oxidative injury induced by H_2_O_2_. F3 at a level of 1 mg/mL could restore the cell death induced by H_2_O_2_ of up to 60% and could alleviate the increase in cellular oxidation (MDA, 8-OHdG, and ROS) induced by H_2_O_2_. Moreover, F3 could restore the activities of antioxidant enzymes suppressed by H_2_O_2_. In conclusion, F3 obtained using supercritical carbon dioxide fractionation could significantly increase the antioxidant capacity of *P. ginseng* extract. The antioxidant capacity was highly correlated with the concentration of F3.

## 1. Introduction

The retinal pigment epithelium is the pigmented cell layer that constitutes the outer blood-retinal barrier. The combination of light and high oxygen consumption puts the retinal pigment epithelial cells under a strong oxidative stress. The oxidative damage of retinal pigment epithelia is known to be involved in the pathogenesis of age-related macular degeneration [1].

*Panax ginseng* is a widely used traditional Chinese medicine for the treatment of various diseases, such as cancer [2], diabetes [3], and cardiovascular diseases [4,5]. Because of its antioxidant activities [6,7,8], *P. ginseng* has been used for improving the immune system [9] and central nervous system functions, and relieving stress. Ginsenosides, also known as ginseng saponins, have been recognized as the principal components of *P. ginseng* and they are responsible for the therapeutic and pharmacological effects of ginseng [10]. Approximately 40 ginsenoside compounds have been identified and they can be categorized into three groups based on their structures: protopanaxadiol, protopanaxatriol, and decglycoslated ginsenosides [11]. Studies found that the functionalities of ginseng varied due to the diversities of ginsenoside structures. For instance, deglycosylated ginsenosides are generally easier to be absorbed and thus exhibit more bioactive functions [12]. In addition, studies demonstrated that deglycosylated ginsenosides could exert stronger anti-inflammation, antidepressant-like effects, and anti-cancer activity than protopanaxadiol and protopanaxatriol ginsenosides [13,14,15].

One important function of ginseng is its antioxidative property in protecting cells from reactive oxygen species (ROS)-induced damage. ROS are generated as byproducts during mitochondrial metabolism and excessive ROS can induce massive oxidation of proteins, lipid, and DNA, leading to cell death. Certain body tissues are especially prone to oxidative damage. For example, the ROS formation caused by the combination of focused light and oxygenated blood conditioned the retinal pigment epithelium of the eye to a constant oxidative stress [16]. The retinal pigment epithelium is the pigmented cell layer that constitutes the outer blood-retinal barrier and it is easily affected by oxidative stress [17], which is believed to be involved in the formation of age-related macular degeneration and can lead to blindness [18]. Despite the fact that a large number of studies have evaluated the protective effects of ginseng against free radical damage to various tissues, such as those of the cardiovascular, liver, and nervous system [19], the effects of different extractions of *P. ginseng* on retinal pigment epithelium is still unclear. Therefore, the aim of this study was to explore the protective effects of the different fractions of *P. ginseng* in retina cells against oxidative stress.

## 2. Results

### 2.1. Cytotoxicity of H_2_O_2_ on ARPE-19 Cells

**Figure 1:**
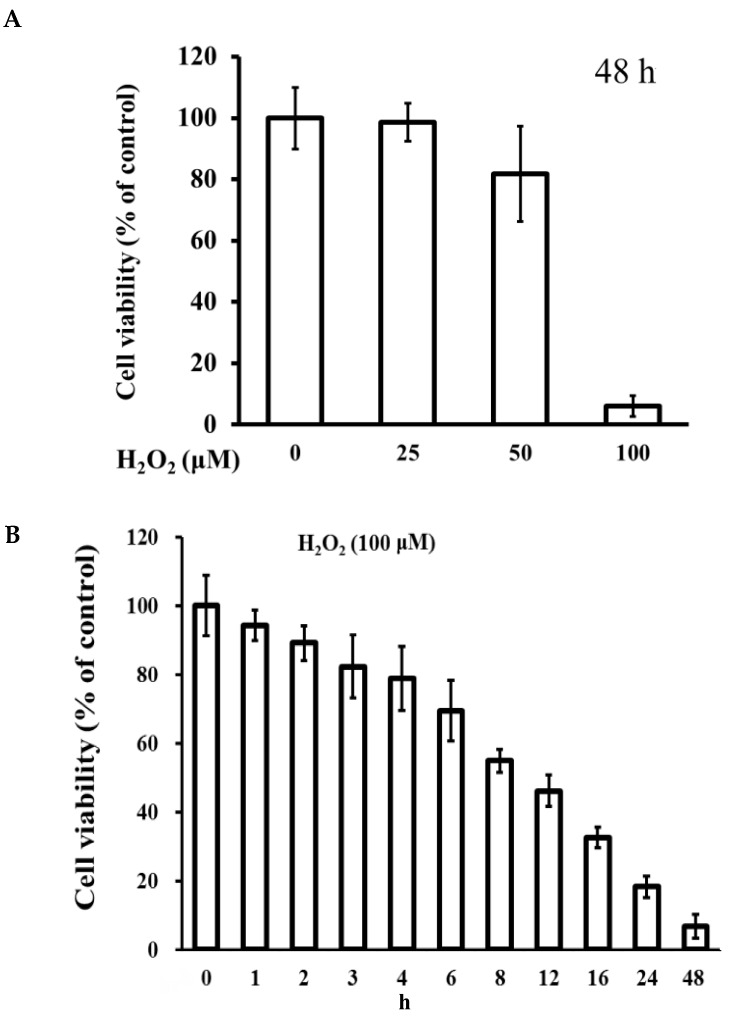
Cytotoxic effect of H_2_O_2_ on adult retinal pigment epithelium-19 (ARPE-19) cells. Cell viability of ARPE-19 cells, following different concentrations of H_2_O_2_ exposure, measured by trypan blue exclusion test. Dose (**A**); and (**B**) time effect of H_2_O_2_ (100 μM) on ARPE-19 cells. Results are expressed as percentages of control, and each value represents the mean ± SD of three independent experiments.

In the present study, we examined whether extractive fractions of *P. ginseng* C. A. Meyer could inhibit oxidative stress-induced cytotoxicity through a reduction of intracellular ROS. To test this hypothesis, we first analyzed the cytotoxicity of ARPE-19 cells under different treatments. As expected, the treatment of H_2_O_2_ significantly induced cytotoxicity in ARPE-19 cells in comparison with normal control cells in both dose and time-dependent manners (Figure 1A,B). The results showed that 100 μM of H_2_O_2_ could reduce the cell viability over 90% after exposure to H_2_O_2_ for 48 h.

### 2.2. Effects of P. ginseng Supercritical CO_2_ Fractions on Cell Viability of H_2_O_2_-Induced Cytotoxicity in ARPE-19 Cells

**Figure 2:**
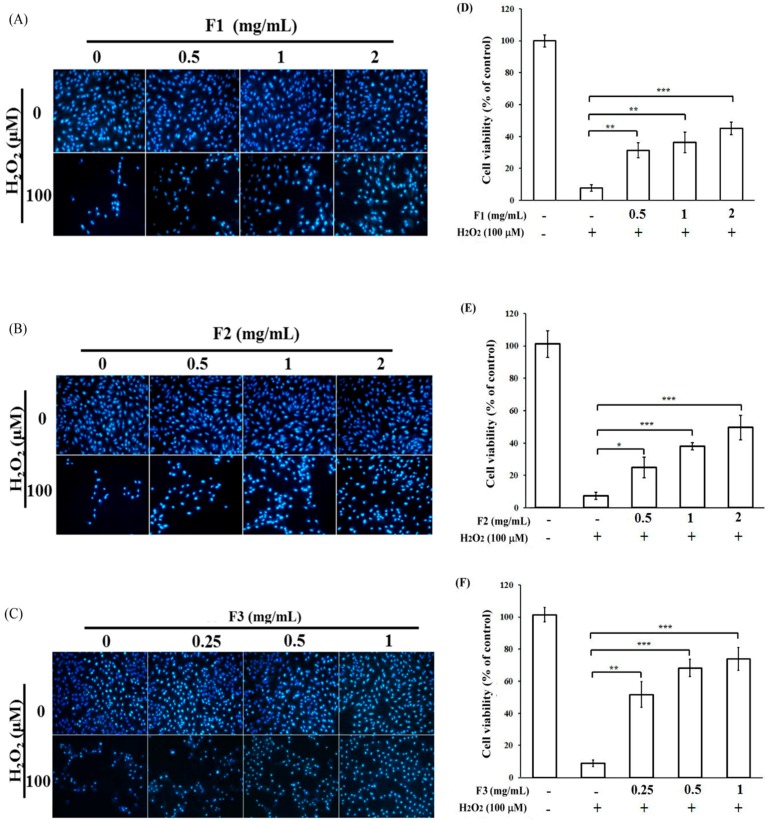
Effects of *P. ginseng* supercritical CO_2_ extracted fractions on cell viability of H_2_O_2_-induced cytotoxicity in ARPE-19 cells. ARPE-19 cells were treated with or without a series of concentrations of *P. ginseng* supercritical CO_2_ fractions for 2 h, and then treated with H_2_O_2_ for another 24 h. (**A**–**C**) Hoechst 33342 staining; (**D**–**F**) Quantitative analysis of panel (**A**–**C**). * *p* < 0.05, ** *p* < 0.01, *** *p* < 0.001 versus H_2_O_2_ group. Results are expressed as percentages of control, and each value represents the mean ± SD of three independent experiments.

In the subsequent experiment, APRE-19 cells were left untreated or pretreated with various dosages of ginseng supercritical CO_2_ fractions F1, F2, and F3, followed by treatment with H_2_O_2_. The results showed that H_2_O_2_ could reduce the viability of APRE-19 cells below 10% with chromosome condensation (Figure 2). The pretreatment of either F1 or F2 at a level of 2 mg/mL could rescue the cell death induced by H_2_O_2_ only up to 30%–40% accompanied by a reduction of chromosome condensation (Figure 2A,B,D,E). However, the pretreatment of F3 at a level of only 1 mg/mL showed stronger rescued effects of up to 60% on APRE-19 cell death induced by H_2_O_2_, with a significant reduction of chromosome condensation in the presence of H_2_O_2_ (Figure 2C,F). These results indicated that the F3 fraction could be an important component of *P. ginseng* in preventing cell death induced by oxidative stress.

### 2.3. Effects of P. ginseng F3 Fraction on Cellular Oxidation Induced by H_2_O_2_ in ARPE-19 Cells

**Figure 3:**
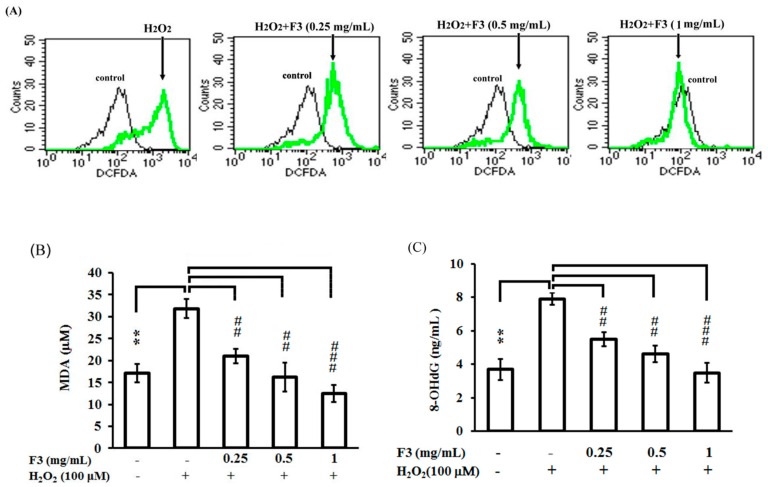
Effects of *P. ginseng* supercritical CO_2_ extracted fraction F3 on H_2_O_2_-induced cellular oxidation in ARPE-19 cells. ARPE-19 cells were pretreated with or without a series of concentrations of F3 for 2 h, and then treated with 100 μM H_2_O_2_ for another 24 h. (**A**) Intracellular reactive oxygen species levels were measured by flow cytometry analysis; (**B**) malondialdehyde (MDA); and (**C**) 8-hydroxydeoxyguanosine (8-OHdG) contents were measured on a microplate reader (VersaMax). MDA and 8-OHdG levels in the H_2_O_2_ group increased significantly compared with the untreated group. F3 attenuated the H_2_O_2_-induced changes in MDA and 8-OHdG. ** *p* < 0.01 versus untreated group. ^##^ *p* < 0.01, ^###^ *p* < 0.001 versus H_2_O_2_ group. Each value represents the mean ± SD of three independent experiments.

To confirm how F3 rescued APRE-19 cells from H_2_O_2_-induced cell death, ARPE-19 cells were either untreated or pretreated with F3 fraction for 2 h followed by the administration of H_2_O_2_ for another 24 h, the cells were then collected and the contents of ROS were determined. The results showed the F3 fraction could effectively reduce the generation or accumulation of H_2_O_2_ in a dose-dependent manner (Figure 3A). Furthermore, both the lipid oxidation (MDA assay) (Figure 3B) and the DNA damage (8-OHdG assay) (Figure 3C) induced by H_2_O_2_ were also significantly attenuated in the presence of F3 in a dose-dependent manner. These results suggested that F3 might be able to prevent ARPE-19 cell death induced by H_2_O_2_ via attenuating the generation or accumulation of ROS and the subsequent lipid and DNA damages.

### 2.4. Effects of P. ginseng F3 Fraction on Antioxidant Enzymes Activities Induced by H_2_O_2_ in ARPE-19 Cells

**Figure 4:**
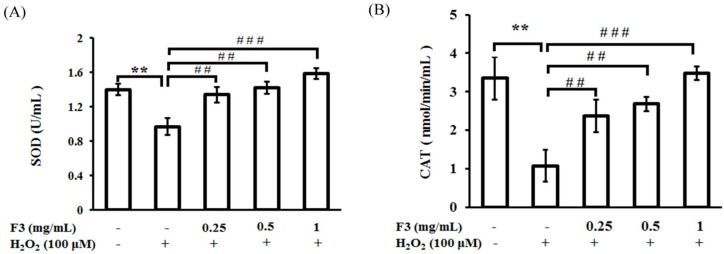

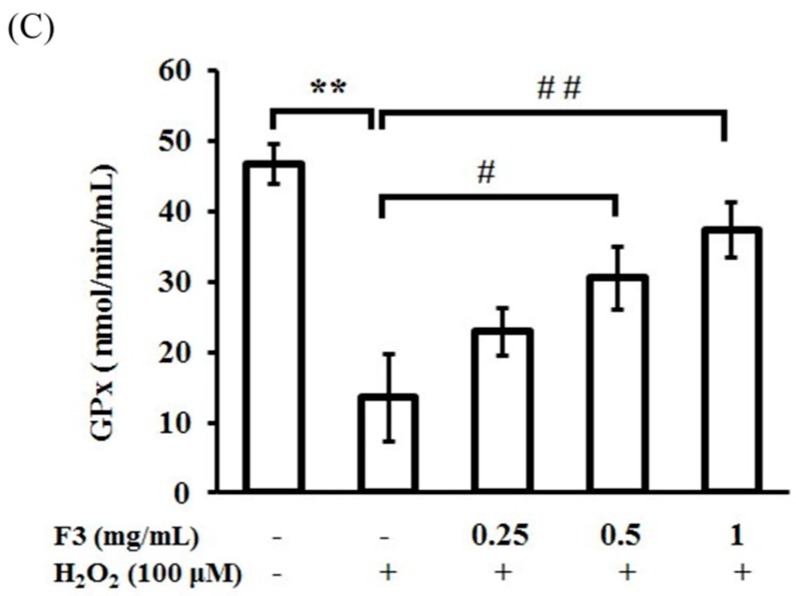
Effect of *P. ginseng* supercritical CO_2_ extracted fraction F3 on cellular antioxidant enzymes: (**A**) superoxide dismutase (SOD); (**B**) catalase (CAT); and (**C**) glutathione peroxidase (GPx) activities in ARPE-19 cells subjected to H_2_O_2_-induced oxidative stress. ARPE-19 cells were pretreated with or without a series of concentrations of F3 for 2 h, and then treated with 100 μM H_2_O_2_ for another 24 h. Antioxidant enzymes activities were determined using commercially available kits following the manufactures’ instructions. ** *p* < 0.01 versus untreated group, ^#^ *p* < 0.05, ^##^ *p* < 0.01, ^###^ *p* < 0.001 versus H_2_O_2_ group. Each value represents the mean ± SD of three independent experiments.

In the next experiment, to confirm the relationships between ROS reduction and F3, we continued to analyze the effects of F3 in regulating the activity of antioxidant enzymes in the presence of H_2_O_2_. The results showed that the addition of H_2_O_2_ significantly decreased the activities of superoxide dismutase, catalase, and glutathione peroxidase in ARPE-19 cells (Figure 4). The pretreatment with F3 effectively restored the activities of all three tested antioxidant enzymes back to the normal levels even in the presence of H_2_O_2_. These results suggested that F3 might increase the activity of antioxidant enzymes to avoid the oxidative stress and subsequent cellular damage induced by H_2_O_2_.

## 3. Discussion

In this study, we investigated the antioxidation ability of supercritical CO_2_ extracted fractions of the root powder of *P. ginseng* C. A. Meyer and explored the reasons for the high antioxidative activity exhibited by F3. Results from our previous study [20] showed that the E, R, F1, and F2 fractions were composed mainly of protopanaxadiol and protopanaxtriol and F3 contained a higher level of deglycosylated ginsenosides comparing with other fractions of *P. ginseng*. Since deglycosylated ginsenosides are involved in the oxidative stress-resistant process, it is reasonable to infer that the highest antioxidation ability of F3 could contribute to the presence of deglycosylated ginsenosides. Both protopanaxadiol and protopanaxtriol exhibited minor effects in protecting ARPE-19 cells from H_2_O_2_-induced oxidative stress. In addition, deglycosylated ginsenosides showed the strongest protective effects in ARPE-19 cells against cytotoxicity induced by H_2_O_2_. Furthermore, deglycosylated ginsenosides recovered the activity of antioxidant enzymes, including the H_2_O_2_-induced oxidative damage, catalase, and glutathione peroxidase, suggesting that a possible pathway of deglycosylated ginsenosides of *P. ginseng* in reducing oxidative stress.

Under normal physiological conditions, cells need to maintain an optimal level of ROS to manipulate a proper signal transduction and to avoid damage by free radicals. The free radical theory of aging suggests that aging and its related disorders are caused by the continuous accumulation of ROS, which can react with multiple molecules, such as DNA, protein, and lipids. In the retinal system, the oxidative damage from ROS induced by the combination of focused light and oxygen in retina epithelial cells is involved in the pathogenesis of age-related macular degeneration [1]. Woo et al. showed that curcumin could protect retinal pigment epithelial cells against oxidative stress by inducing the levels of phase II enzymes heme oxygenase-1 [21]. Wankun et al. also demonstrated a protective effect of paeoniflorin against oxidative stress via the mitogen-activated protein kinase pathways in retinal pigment epithelial cells [22]. Furthermore, Li et al. showed that astaxanthin could protect retinal pigment epithelial cells from oxidative stress via upregulating phase II enzymes in accompanied with the activation of PI3K/Akt [23]. These results showed that the upregulation of metabolic enzymes is a major pathway for protection against oxidative stress. However, these reports did not mention the effects of these compounds in the activities of these metabolic enzymes. Conversely, our results clearly showed that an activity induction of metabolic enzymes, including superoxide dismutase, catalase, and glutathione peroxidase by supercritical CO_2_ extracted fraction F3 of *P. ginseng* in the condition of oxidative stresses primed by H_2_O_2_ (Figure 4). Therefore, the role of F3 in the pathogenesis of oxidative stress-related ocular diseases warrants further investigation.

Ginseng is a popular medicinal herb that contains plenty of its active constituent ginsenosides. Some components of ginsenosides, for example, Rb1, Rg1, and Re, might activate certain antioxidant enzymes and thereby offer protection against cell death. An in vitro study of oxidative stress and cell death in brain cells showed that the protective effects of *P. notoginseng* in astrocytes were associated with attenuation of ROS accumulation. Such effect was involved the activation of Nrf2 (nuclear translocation) and upregulation of downstream antioxidant systems, which included heme oxygenase-1 and glutathione S-transferase pi 1 [24]. Betts et al. described that Rb1 ginsenoside could potentially induce ARPE-19 proliferation and thereby reduced the release of vascular endothelial growth factor [25]. When vascular endothelial growth factor is overexpressed, it can contribute to vascular disease in the retina of the eye. Furthermore, Li et al. demonstrated that Rg1 ginsenoside could protect retinal pigment epithelial cells against hypoxia and cobalt chloride assaults [26], indicating a protective role of *P. ginseng* in retinal pigment epithelial cells. In the present study, we showed that H_2_O_2_ could strongly induce intracellular ROS generation and mortality of ARPE-19 cells, and the effect could significantly be alleviated by the treatment of F3 (Figure 3).

## 4. Materials and Methods

### 4.1. Materials

*P. ginseng* C. A. Meyer was obtained from Wah Hong Ginseng Company, Kaohsiung City, Taiwan. 2′,7′-dichlorodihydrofluorescein diacetate (H_2_DCFDA), fetal bovine serum, H_2_O_2_, bisBenzimide H33342 trihydrochloride, and trypan blue solution were purchased from Sigma (St. Louis, MO, USA). Dulbecco’s modified Eagle’s medium was purchased from Invitrogen. Lipid Peroxidation (malondialdehyde, MDA) Colorimetric/Fluorometric Assay Kit, Superoxide Dismutase (SOD) Activity Assay Kit, Catalase Activity Colorimetric/Fluorometric Assay Kit and Glutathione Colorimetric Assay Kit were purchased from Biovision (Milpitas, CA, USA). DNA damage ELISA kit (8-hydroxydeoxyguanosine, 8-OHdG) was purchased from Enzo Life Sciences, Inc. (Farmingdale, NY, USA).

### 4.2. Extraction and Fractionation

Ethanolic extracts from *P. ginseng* C. A. Meyer was fractionated into R, F1, F2, and F3 according to the method described in our previous work [20]. The root powder of *P. ginseng* was extracted twice using 85% ethanol at a ratio of 1:2 (*w*/*v*) for 24 h per extraction. The operating conditions of the fractionation were set at a temperature of 45 °C, a system pressure of 30 MPa, a flow rate of CO_2_ at 4 mL/min, and a sample inflow rate of 1 mL/min. A series of separation vessels was operated at pressures of 30, 25, 20 and 15 MPa to obtain three fractions (F1, F2, and F3) and a residue (R). The obtained samples were freeze-dried and stored at −20 °C until use.

### 4.3. Cell Culture and Viability Assay

ARPE-19 cells (ATCC^®^ CRL-2302), originated from human retinal pigment epithelial cells, were cultured in Dulbecco’s modified Eagle’s medium supplemented with 10% (*v*/*v*) heat-inactivated fetal bovine serum and 100 U/mL penicillin and streptomycin. The cells were incubated at 37 °C in a humidified 5% CO_2_ atmosphere. The cells were plated in 12-well culture dishes at a density of 2 × 10^5^ cells/mL and incubated overnight. Cells were pretreated with each ginseng fraction (0.25–2 mg/mL) for 2 h and then stimulated with H_2_O_2_ (100 μM) for indicated time points. The number of viable cells was determined by the trypan blue dye exclusion method. Cell viability was expressed as a percentage of the value in the control.

### 4.4. Hoechst 33342 Staining

Hoechst 33342 staining was used to observe changes of nuclei morphology of ARPE-19 cells after H_2_O_2_ treatment. After treatment with the indicated concentrations of H_2_O_2_ and fractions, cells were stained with 10 µM Hoechst 33342 for 15 min at room temperature. The stained cells were rinsed three times with phosphate-buffered saline and then observed using a fluorescence microscope with standard excitation filters.

### 4.5. Measurement of Intracellular Reactive Oxygen Species

Intracellular ROS levels were measured using 2′,7′-dichlorodihydrofluorescein diacetate (H_2_DCFDA), which is converted into a non-fluorescent derivative by intracellular esterases. ARPE-19 cells were plated at a density of 5 × 10^5^ cells per 6-well plates and incubated overnight. Cells were pretreated with each F3 fraction (0.25–1 mg/mL) for 2 h and then exposed to 100 μM H_2_O_2_ for 24 h. The cells were incubated with H_2_DCFDA at 5 μM for 30 min at 37 °C. At the end of incubation, the cells were washed three times with phosphate-buffered saline and harvested. Fluorescence was measured with a FACSCan flow cytometer. ROS levels were measured using excitation and emission wavelengths of 488 nm and 525 nm, respectively. Enzyme-linked immunosorbent assays (ELISA) were used to measure 8-OH-dG levels, according to the manufacturer’s instructions. MDA was measured at a wavelength of 532 nm by reacting with thiobarbituric acid (TBA) and the results were expressed as thiobarbituric acid reactive substances (TBARS).

### 4.6. Determination of Superoxide Dismutase, Catalase, and Glutathione Peroxidase Activities

ARPE-19 cells were plated at a density of 5 × 10^5^ cells per 6-well plates and incubated overnight. Cells were pretreated with each F3 fraction (0.25–1 mg/mL) for 2 h and then exposed to 100 μM H_2_O_2_ for 24 h. After cultured, the cells were washed with phosphate-buffered saline. The cells were then scraped from the plates into ice-cold phosphate-buffered saline and homogenized. Intracellular superoxide dismutase, catalase, and glutathione peroxidase activities were determined using commercially available kits and measured spectrophotometrically at 450, 520 and 415 nm, respectively.

### 4.7. Statistical Analysis

All data are expressed as mean ± standard deviation of at least three independent experiments. One-way analysis of variance with Duncan’s multiple range tests were used for comparisons. Two-tailed *p* < 0.05 was considered statistically significant.

## 5. Conclusions

In conclusion, the present study showed that F3, but not F1 and F2, from supercritical CO_2_ extracted fractions of root powder of *P. ginseng* C. A. Meyer, could effectively protect ARPE-19 cells against H_2_O_2_-induced cell death via a reduction in oxidative stress. Further results demonstrated that F3 could potentially increase the activity of phase II enzymes, including superoxide dismutase, catalase, and glutathione peroxidase in the presence of H_2_O_2_. Therefore, the confirmative component of F3 responsible for the strong protective effect of retinal pigment epithelial cells should be elucidated further.

## Abbreviations

8-OHdG	8-hydroxydeoxyguanosine
ARPE-19	adult retinal pigment epithelium-19
CAT	catalase
CO_2_	carbon dioxide
H_2_DCFDA	2′,7′-dichlorodihydrofluorescein diacetate
DNA	deoxyribonucleic acid
ELISA	enzyme-linked immunosorbent assay
GPx	glutathione peroxidase
H_2_O_2_	hydrogen peroxide
MDA	malondialdehyde
ROS	reactive oxygen species
SD	standard deviation
SOD	superoxide dismutase
TBA	thiobarbituric acid
TBARS	thiobarbituric acid reactive substances
